# Supplementary material for: Correlation between Molecular Docking and the Stabilizing Interaction of HOMO-LUMO: Spirostans in CHK1 and CHK2, an In Silico Cancer Approach
Source: Int J Mol Sci. 2024 Aug 6;25(16):8588. doi: 10.3390/ijms25168588 (PMC11354435; doi:10.3390/ijms25168588)
Supplement: Supplementary file 1 [file ijms-25-08588-s001.zip › ijms-3036896-supplementary-done.pdf]

International Journal of *Molecular Sciences*

MDPI

Article

Correlation molecular docking and of stabilizing interaction HOMO-LUMO: Spirostan sapogenins in CHK1 and CHK2, an *in silico* cancer approach

Antonio Rosales-López [1], Guiee N. López-Castillo [1,2], Jesús Sandoval-Ramírez [1,2], Joel L. Terán [3,*] , Alan Carrasco-Carballo [1,3,4*]

[1] Laboratorio de Elucidación y Síntesis en Química Orgánica, BUAP, Puebla, Pue., México; alan.carrascoc@correo.buap.mx
[2] Laboratorio de Modificación y Síntesis en Productos Naturales, FCQ, BUAP, Puebla, México; jesus.sandoval@correo.buap.mx
[3] Centro de Química, Instituto de Ciencias, BUAP, Puebla, Pue., México; joel.teran@correo.buap.mx
[4] CONAHCYT, LESQO, ICUAP, BUAP, Puebla, Pue., México; alan.carrasco@conahcyt.mx
[*] Correspondence: alan.carrascoc@correo.buap.mx (A.C.C.); joel.teran@correo.buap.mx (J.T)

Supplementary Material

Table S1. Interactional analysis of Sp with CHK1 2
Table S2. Interactional analysis of Sp with CHK2 3

Table S3. HOMO-LUMO analysis in catalytic site for selective inhibitors for CHK1 4
Table S4. HOMO-LUMO analysis in catalytic site for selective inhibitors for CHK2 6
Table S5. List of designed spirostans 8
Figure S1: Ligand-protein contacts, stability, RMSF per ligand, SASA, PSA, torsions and contacts as a function of time with CHK1 for a) CCT241533; b) Sp001; c) Sp137 13
Figure S2: Ligand-protein contacts, stability, RMSF per ligand, SASA, PSA, torsions and contacts as a function of time with CHK2 for a) PD407824; b) Sp013; c) Sp137. 19
Figure S3. Representative RMSD at 150 ns in CHK1 to CCT241533 and Sp137 25

Table S1. Interactional analysis of Sp with CHK1

Sapogenin	Charged(-)	Charged(+)	Glycine	Hydrophobic	Polar	H-Bond
Sp1	Glu85, **Glu91**, Asp94, **Asp148**	Lys38	**Gly16**, **Gly89**, **Gly90**	**Leu15**, **Val23**, Ala36, Val68, Leu84, **Tyr86**, **Cys87**, **Leu137**	**Gln13**, **Ser88**, **Ser147**	Tyr86, **Asp148**
Sp3	Glu85, **Glu91**, Asp94, **Glu134**, **Asp148**	Lys38	**Gly16**, **Gly89**, **Gly90**	**Leu15**, **Val23**, Ala36, Val68, Leu84, **Tyr86**, **Cys87**, **Leu137**	**Gln13**, **Ser88**, **Ser147**	Tyr86, **Asp148**
Sp24	Glu85, **Glu91**, Asp94, **Asp148**	Lys38	**Gly16**, **Gly89**, **Gly90**	**Leu15**, **Val23**, Ala36, Val68, Leu84, **Tyr86**, **Cys87**, **Leu137**	**Gln13**, **Ser88**, **Ser147**	Tyr86, **Asp148**
Sp37	Glu85, **Glu91**, Asp94, **Asp148**	Lys38	**Gly16**, **Gly89**, **Gly90**	**Leu15**, **Val23**, Ala36, Val68, Leu84, **Tyr86**, **Cys87**, **Leu137**	**Gln13**, **Ser88**, **Ser147**	Tyr86, **Asp148**
Sp53	Glu85, **Glu91**, Asp94	-	**Gly16**, **Gly89**, **Gly90**	**Leu15**, **Val23**, Ala36, **Tyr86**, **Cys87**, **Leu137**	**Gln13**, **Ser88**	Cys87
Sp67	Glu85, Glu91, Asp94	-	**Gly16**, **Gly89**, **Gly90**	**Leu15**, **Val23**, Ala36, **Tyr86**, **Cys87**, **Leu137**	**Gln13**, **Ser88**	Cys87
Sp97	Glu85, **Glu91**, Asp94, **Asp148**	Lys38	**Gly16**, **Gly89**, **Gly90**	**Leu15**, **Val23**, Ala36, Val68 Leu84, **Tyr86**, **Cys87**, **Leu137**,	**Gln13**, **Ser88**, **Ser147**	Tyr86, **Asp148**
Sp101	**Glu91**, Asp94	-	**Gly16**, **Gly89**, **Gly90**	**Leu15**, **Val23**, Leu25, Ala36, **Tyr86**, **Cys87**, **Leu137**, Leu138	**Gln13**, **Ser88**	**Gln13**
Sp109	**Glu91**, Asp94	-	**Gly16**, **Gly89**, **Gly90**	**Leu15**, **Val23**, Ala36, **Tyr86**, **Cys87**, **Leu137**	**Gln13**, Thr14, **Ser88**	**Glu91**
Sp131	Glu17, **Glu91**, Asp94	-	**Gly16**, **Gly89**, **Gly90**	**Leu15**, Leu25, **Tyr86**, **Cys87**	**Gln13**, **Ser88**	Leu15, Asp94
Sp137	Glu85, **Glu91**, Asp94	-	**Gly16**, **Gly89**, **Gly90**	**Leu15**, **Val23**, Ala36, **Tyr86**, **Cys87**, **Leu137**, Leu138	**Gln13**, **Ser88**	**Glu91**
Sp142	Glu85, **Glu91**	-	**Gly16**, **Gly89**, **Gly90**	**Leu15**, **Val23**, Ala36, Val68, Leu84, **Tyr86**, **Cys87**, **Leu137**, Leu138	**Gln13**, Thr14, **Ser88**	Cys87

Sp154	Glu85, **Glu91**, Asp94	-	**Gly16**, **Gly90**	**Leu15**, **Val23**, Ala36, Leu84, **Tyr86**, **Cys87**, **Leu137**, Leu138	**Gln13**, **Ser88**	-
Sp155	Glu85, **Glu91**, Asp94	-	**Gly16**, **Gly90**	**Leu15**, **Val23**, Ala36, Leu84, **Tyr86**, **Cys87**, **Leu137**, Leu138	**Gln13**, **Ser88**	-

Table S2. Interactional analysis of Sp with CHK2

Sapogenin	Charged(-)	Carged(+)	Gly	Hydrophobic	Polar	H-Bond
Sp5	**Glu305**, **Glu308**, **Asp311**	Lys245	**Gly227**, **Gly306**, **Gly307**	**Leu226**, **Leu303**, **Met304**	**Gln358**	Asp311
Sp13	**Glu305**, **Glu308**, **Asp311**	Lys245	**Gly227**, **Gly306**, **Gly307**	**Leu226**, **Leu303**, **Met304**	**Gln358**	Asp311
Sp14	**Glu305**, **Glu308**, **Asp311**	Lys245	**Gly227**, **Gly306**, **Gly307**	**Leu226**, **Leu303**, **Met304**	**Gln358**	Asp311
Sp16	**Glu305**, **Glu308**, **Asp311**	Lys245	**Gly227**, **Gly306**, **Gly307**	**Leu226**, **Leu303**, **Met304**	**Gln358**	Asp311
Sp28	**Glu305**, **Glu308**, **Asp311**	Lys245	**Gly227**, **Gly306**, **Gly307**	**Leu226**, Leu236, **Leu303**, **Met304**	**Gln358**	**Glu308**, Lys245
Sp35	**Glu305**, **Glu308**, **Asp311**	Lys245	**Gly227**, **Gly306**, **Gly307**	**Leu226**, **Val234**, Leu236, **Ala247**, **Leu303**, **Met304**, **Leu354**	**Gln358**, Thr367	-
Sp41	**Glu305**, **Glu308**, **Asp311**	Lys245	**Gly227**, **Gly306**, **Gly307**	**Leu226**, **Leu303**, **Met304**	**Gln358**	Asp311
Sp42	**Glu305**, **Glu308**, **Asp311**	Lys245	**Gly227**, **Gly306**, **Gly307**	**Leu226**, **Leu303**, **Met304**	**Gln358**	Asp311
Sp44	**Glu305**, **Glu308**, **Asp311**	Lys245	**Gly227**, **Gly306**, **Gly307**	**Leu226**, **Leu303**, **Met304**	**Gln358**	Asp311
Sp45	**Glu305**, **Glu308**, **Asp311**	Lys245	**Gly227**, **Gly306**, **Gly307**	**Leu226**, **Leu303**, **Met304**	**Gln358**	Asp311

Sp65	Glu302, **Glu305**, **Glu308**, **Asp311**	-	**Gly227**, **Gly306**, **Gly307**	**Leu226**, **Val234**, **Ala247**, Ile286, Leu301, **Leu303**, **Met304**, **Leu354**	Thr367	-
Sp106	**Glu305**, **Glu308**, **Asp311**	Lys245	**Gly227**, **Gly306**, **Gly307**	**Leu226**, Leu236, **Leu303**, **Met304**	Ser228, **Gln358**	Lys245, Met304
Sp137	Glu302, **Glu305**, **Glu308**, **Asp311**	-	**Gly227**, **Gly306**, **Gly307**	**Leu226**, **Val234**, **Ala247**, Ile286, Leu301, **Leu303**, **Met304**, **Leu354**, Leu355	-	**Glu308**
Sp139	**Glu305**, **Glu308**, **Asp311**	-	**Gly227**, **Gly306**, **Gly307**	**Leu226**, **Val234**, **Ala247**, **Leu303**, **Met304**, **Leu354**	**Gln358**, Thr367	Gln358

Table S3. HOMO-LUMO analysis in catalytic site for selective inhibitors for CHK1.

	**HOMO$_P$**	**LUMO$_P$**	**HOMO$_P$-LUMO$_P$**		**Sp137**	**Sp1**	**Sp3**	**Sp24**	**Sp37**	**Sp53**	**Sp67**	**Sp97**	**Sp101**	**Sp109**	**Sp131**	**Sp142**	**Sp154**	**Sp155**
**Gln13**	-0.2397	-0.0307	-0.2090	**HOMO$_P$-LUMO$_{Sp}$**	-0.1989	-0.2687	-0.2688	-0.2635	-0.2214	-0.3048	-0.3046	-0.2637	-0.2283	-0.3026	-0.2975	-0.1855	-0.1974	-0.1973
				**HOMO$_{Sp}$-LUMO$_P$**	-0.1953	-0.1966	-0.1965	-0.2009	-0.1987	-0.2166	-0.2172	-0.2008	-0.2028	-0.2159	-0.2100	-0.2138	-0.1979	-0.1978
**Val23**	-0.2331	-0.0270	-0.2061	**HOMO$_P$-LUMO$_{Sp}$**	-0.1924	-0.2621	-0.2622	-0.2570	-0.2148	-0.2982	-0.2980	-0.2571	-0.2217	-0.2960	-0.2909	-0.1789	-0.1908	-0.19075
				**HOMO$_{Sp}$-LUMO$_P$**	-0.1990	-0.2003	-0.2002	-0.20459	-0.2024	-0.2202	-0.2209	-0.2045	-0.2065	-0.2195	-0.2137	-0.2175	-0.2016	-0.2015
**Ala36**	-0.2377	-0.0278	-0.2098	**HOMO$_P$-LUMO$_{Sp}$**	-0.1969	-0.2666	-0.2668	-0.2615	-0.2194	-0.30280	-0.3026	-0.2617	-0.2263	-0.3005	-0.2955	-0.1835	-0.1954	-0.1953
				**HOMO$_{Sp}$-LUMO$_P$**	-0.1982	-0.1994	-0.1994	-0.2037	-0.2015	-0.2194	-0.2200	-0.2037	-0.2057	-0.2187	-0.2128	-0.2166	-0.2008	-0.2006
**Glu85**	-0.0241	0.0851	-0.1092	**HOMO$_P$-LUMO$_{Sp}$**	0.0166	-0.0530	-0.0532	-0.0479	-0.0058	-0.0891	-0.0890	-0.0480	-0.0127	-0.0869	-0.0819	0.0300	0.0182	0.0182
				**HOMO$_{Sp}$-**	-0.3112	-0.3125	-0.3124	-0.3167	-0.3146	-0.3324	-0.3331	-0.3167	-0.3187	-0.3317	-0.3259	-0.3297	-0.3138	-0.3137

				**$LUMO_P$**														
**Try86**	-0.2232	-0.0230	-0.2002	**$HOMO_P$-$LUMO_{Sp}$**	-0.1825	-0.2522	-0.2524	-0.2471	-0.2049	-0.2883	-0.2882	-0.2472	-0.2119	-0.2861	-0.2811	-0.1691	-0.1809	-0.1808
				**$HOMO_{Sp}$-$LUMO_P$**	-0.2030	-0.2042	-0.2041	-0.2085	-0.2063	-0.2242	-0.2248	-0.2085	-0.2105	-0.2235	-0.2176	-0.2214	-0.2055	-0.2054
**Cys87**	-0.2508	-0.0314	-0.2194	**$HOMO_P$-$LUMO_{Sp}$**	-0.2101	-0.2798	-0.2800	-0.2747	-0.2325	-0.3159	-0.3158	-0.2748	-0.2395	-0.3137	-0.3087	-0.1967	-0.2085	-0.2084
				**$HOMO_{Sp}$-$LUMO_P$**	-0.1946	-0.1958	-0.1957	-0.2001	-0.1979	-0.21584	-0.2164	-0.2001	-0.2021	-0.2151	-0.2092	-0.2130	-0.1972	-0.1970
**Ser88**	-0.2593	-0.0185	-0.2407	**$HOMO_P$-$LUMO_{Sp}$**	-0.2186	-0.2883	-0.2884	-0.2832	-0.2410	-0.3244	-0.3242	-0.2833	-0.2479	-0.3222	-0.3171	-0.2051	-0.2170	-0.2169
				**$HOMO_{Sp}$-$LUMO_P$**	-0.2075	-0.2087	-0.2086	-0.2130	-0.2108	-0.2287	-0.2293	-0.2129	-0.2150	-0.2280	-0.2221	-0.2259	-0.2100	-0.2099
**Gly89**	-0.2621	-0.0410	-0.2211	**$HOMO_P$-$LUMO_{Sp}$**	-0.2214	-0.2911	-0.2913	-0.2860	-0.2438	-0.3272	-0.3271	-0.2861	-0.2508	-0.3250	-0.3200	-0.2080	-0.2198	-0.2198
				**$HOMO_{Sp}$-$LUMO_P$**	-0.1850	-0.1862	-0.1861	-0.1905	-0.1883	-0.2062	-0.2068	-0.1905	-0.1925	-0.2055	-0.1996	-0.2034	-0.1875	-0.1874
**Gly90**	-0.2696	-0.0175	-0.2521	**$HOMO_P$-$LUMO_{Sp}$**	-0.2289	-0.2986	-0.2988	-0.2935	-0.2513	-0.3347	-0.3345	-0.2936	-0.2582	-0.3325	-0.3274	-0.2154	-0.2273	-0.2272
				**$HOMO_{Sp}$-$LUMO_P$**	-0.2085	-0.2098	-0.2097	-0.2140	-0.2119	-0.2297	-0.2304	-0.2140	-0.2160	-0.2290	-0.2232	-0.2270	-0.2111	-0.2110
**Asp94**	-0.0385	0.1099	-0.1484	**$HOMO_P$-$LUMO_{Sp}$**	0.0021	-0.0675	-0.0677	-0.0624	-0.0202	-0.1036	-0.1034	-0.0625	-0.0272	-0.1014	-0.0963	0.0156	0.0037	0.0038
				**$HOMO_{Sp}$-$LUMO_P$**	-0.3360	-0.3372	-0.3371	-0.3415	-0.3393	-0.3572	-0.3578	-0.3415	-0.3435	-0.3565	-0.3506	-0.3544	-0.3385	-0.3384
**Glu134**	-0.0354	0.1078	-0.1432	**$HOMO_P$-$LUMO_{Sp}$**	0.0053	-0.0644	-0.0645	-0.0592	-0.0171	-0.1005	-0.1003	-0.0594	-0.0240	-0.0982	-0.0932	0.0187	0.0068	0.0069

				**$HOMO_{Sp}$-$LUMO_{P}$**	-0.3339	-0.3351	-0.3350	-0.3394	-0.3372	-0.3551	-0.3557	-0.3394	-0.3414	-0.3544	-0.3485	-0.3523	-0.3364	-0.3363
**Leu137**	-0.2352	-0.0237	-0.2115	**$HOMO_{P}$-$LUMO_{Sp}$**	-0.1945	-0.2642	-0.2644	-0.2591	-0.2169	-0.3003	-0.3002	-0.2592	-0.2239	-0.2981	-0.2931	-0.1811	-0.1929	-0.1928
				**$HOMO_{Sp}$-$LUMO_{P}$**	-0.2023	-0.2036	-0.2035	-0.2078	-0.2057	-0.2235	-0.2242	-0.2078	-0.2098	-0.2228	-0.2170	-0.2208	-0.2049	-0.2048

$HOMO_{P}$: Highest Occupied Molecular Orbital of protein; $LUMO_{P}$: Lowest Unoccupied Molecular Orbital of protein; $LUMO_{Sp}$: Lowest Unoccupied Molecular Orbital of spirostan; $HOMO_{Sp}$: Highest Occupied Molecular Orbital of spirostan.

Table S4. HOMO-LUMO analysis in catalytic site for selective inhibitors for CHK2.

	**$HOMO_P$**	**$LUMO_P$**	**$HOMO_P$-$LUMO_P$**		**Sp137**	**Sp5**	**Sp13**	**Sp14**	**Sp16**	**Sp28**	**Sp35**	**Sp41**	**Sp42**	**Sp44**	**Sp45**	**Sp65**	**Sp106**	**Sp139**
**Leu226**	-0.2559	-0.0284	-0.2274	**$HOMO_P$-$LUMO_{Sp}$**	-0.2151	-0.3222	-0.2483	-0.2843	-0.2404	-0.2849	-0.2862	-0.248337	-0.313676	-0.2418	-0.3223	-0.239118	-0.318849	-0.2064
				**$HOMO_{Sp}$-$LUMO_P$**	0.0184	-0.0548	-0.0147	-0.0342	-0.0068	-0.0360	-0.0312	-0.014737	-0.052905	-0.0082	-0.0560	-0.005518	-0.054386	0.0271
**Gly227**	-0.2709	-0.0156	-0.2553	**$HOMO_P$-$LUMO_{Sp}$**	-0.2302	-0.3373	-0.2634	-0.2994	-0.2555	-0.3000	-0.3012	-0.2634	-0.3287	-0.2569	-0.3373	-0.2541	-0.3339	-0.2214
				**$HOMO_{Sp}$-$LUMO_P$**	-0.2104	-0.2311	-0.2138	-0.2105	-0.2090	-0.2123	-0.2075	-0.2150	-0.2291	-0.2123	-0.2323	-0.2152	-0.2306	-0.2159
**Val234**	-0.2552	-0.0242	-0.2310	**$HOMO_P$-$LUMO_{Sp}$**	-0.2145	-0.3216	-0.2477	-0.2837	-0.2398	-0.2843	-0.2855	-0.2476	-0.3130	-0.2411	-0.3216	-0.2384	-0.3182	-0.2057
				**$HOMO_{Sp}$-$LUMO_P$**	-0.2018	-0.2225	-0.2052	-0.2019	-0.2004	-0.2037	-0.1989	-0.2064	-0.2205	-0.2037	-0.2237	-0.2066	-0.2220	-0.2073
**Lys245**	-0.3474	-0.1918	-0.1556	**$HOMO_P$-$LUMO_{Sp}$**	-0.3067	-0.4138	-0.3399	-0.3759	-0.3320	-0.3765	-0.3778	-0.3399	-0.4052	-0.3334	-0.4138	-0.3307	-0.4104	-0.2979
				**$HOMO_{Sp}$-$LUMO_P$**	-0.0342	-0.0548	-0.0376	-0.0342	-0.0327	-0.0360	-0.0312	-0.0388	-0.0529	-0.0360	-0.0560	-0.0390	-0.0543	-0.0397
**Ala247**	-0.2608	-0.0236	-0.2372	**$HOMO_P$-$LUMO_{Sp}$**	-0.2201	-0.3272	-0.2533	-0.2893	-0.2454	-0.2899	-0.2911	-0.2532	-0.3186	-0.2467	-0.3272	-0.2440	-0.3238	-0.2113
				**$HOMO_{Sp}$-$LUMO_P$**	-0.2024	-0.2230	-0.2058	-0.2025	-0.2009	-0.2042	-0.1994	-0.2070	-0.2211	-0.2042	-0.2242	-0.2072	-0.2225	-0.2079
**Leu303**	-0.2575	-0.0201	-0.2374	**$HOMO_P$-$LUMO_{Sp}$**	-0.2168	-0.3239	-0.2500	-0.2860	-0.2421	-0.2866	-0.2878	-0.2499	-0.3153	-0.2434	-0.3239	-0.2407	-0.3204	-0.2080
				**$HOMO_{Sp}$-$LUMO_P$**	-0.2059	-0.2266	-0.2093	-0.2060	-0.2045	-0.2078	-0.2030	-0.2105	-0.2246	-0.2078	-0.2278	-0.2107	-0.2261	-0.2114

**Met304**	-0.2272	-0.0283	-0.1988	**$HOMO_{P}$-$LUMO_{Sp}$**	-0.1864	-0.2935	-0.2197	-0.2557	-0.2117	-0.2562	-0.2575	-0.2196	-0.2850	-0.2131	-0.2936	-0.2104	-0.2901	-0.1777
				**$HOMO_{Sp}$-$LUMO_{P}$**	-0.1977	-0.2183	-0.2010	-0.1977	-0.1962	-0.1995	-0.1947	-0.2023	-0.2163	-0.1995	-0.2195	-0.2025	-0.2178	-0.2032
**Glu305**	-0.0223	0.0738	-0.0961	**$HOMO_{P}$-$LUMO_{Sp}$**	0.0184	-0.0886	-0.0148	-0.0508	-0.0068	-0.0513	-0.0526	-0.0147	-0.0800	-0.0082	-0.0887	-0.0055	-0.0852	0.0271
				**$HOMO_{Sp}$-$LUMO_{P}$**	-0.2999	-0.3205	-0.3033	-0.3000	-0.2984	-0.3017	-0.2969	-0.3045	-0.3186	-0.3017	-0.3217	-0.3047	-0.3201	-0.3054
**Gly306**	-0.2622	-0.0410	-0.2212	**$HOMO_{P}$-$LUMO_{Sp}$**	-0.2215	-0.3286	-0.2547	-0.2907	-0.2468	-0.2913	-0.2925	-0.2547	-0.3200	-0.2482	-0.3286	-0.2454	-0.3252	-0.2127
				**$HOMO_{Sp}$-$LUMO_{P}$**	-0.1850	-0.2057	-0.1884	-0.1851	-0.1835	-0.1868	-0.1820	-0.1896	-0.2037	-0.1869	-0.2069	-0.1898	-0.2052	-0.1905
**Gly307**	-0.2711	-0.0136	-0.2575	**$HOMO_{P}$-$LUMO_{Sp}$**	-0.2304	-0.3375	-0.2636	-0.2996	-0.2557	-0.3002	-0.3014	-0.2636	-0.3289	-0.2571	-0.3375	-0.2543	-0.3341	-0.2216
				**$HOMO_{Sp}$-$LUMO_{P}$**	-0.2124	-0.2331	-0.2158	-0.2125	-0.2109	-0.2142	-0.2094	-0.2170	-0.2311	-0.2142	-0.2343	-0.2172	-0.2326	-0.2179
**Glu308**	-0.0223	0.0762	-0.0985	**$HOMO_{P}$-$LUMO_{Sp}$**	0.0184	-0.0886	-0.0147	-0.0507	-0.0068	-0.0513	-0.0526	-0.0147	-0.0800	-0.0082	-0.0887	-0.0055	-0.0852	0.0271
				**$HOMO_{Sp}$-$LUMO_{P}$**	-0.3023	-0.3230	-0.3057	-0.3024	-0.3008	-0.3041	-0.2993	-0.3069	-0.3210	-0.3042	-0.3242	-0.3071	-0.3225	-0.3078
**Asp311**	-0.0303	0.0831	-0.1134	**$HOMO_{P}$-$LUMO_{Sp}$**	0.0104	-0.0967	-0.0228	-0.0588	-0.0149	-0.0594	-0.0606	-0.0227	-0.0881	-0.0162	-0.0967	-0.0135	-0.0932	0.0191
				**$HOMO_{Sp}$-$LUMO_{P}$**	-0.3091	-0.3298	-0.3125	-0.3092	-0.3077	-0.3110	-0.3062	-0.3137	-0.3278	-0.3110	-0.3310	-0.3139	-0.3293	-0.3147
**Leu354**	-0.2531	-0.0292	-0.2239	**$HOMO_{P}$-$LUMO_{Sp}$**	-0.2124	-0.3195	-0.2456	-0.2816	-0.2377	-0.2822	-0.2835	-0.2456	-0.3109	-0.2391	-0.3195	-0.2364	-0.3161	-0.2036
				**$HOMO_{Sp}$-**	-0.1968	-0.2174	-0.2001	-0.1968	-0.1953	-0.1986	-0.1938	-0.2013	-0.2154	-0.1986	-0.2186	-0.2016	-0.2169	-0.2023

				**$LUMO_P$**														
**Gln358**	-0.2555	-0.0250	-0.2304	**$HOMO_P$-$LUMO_{Sp}$**	-0.2147	-0.3218	-0.2480	-0.2840	-0.2400	-0.2845	-0.2858	-0.2479	-0.3133	-0.2414	-0.3219	-0.2387	-0.3184	-0.2060
				**$HOMO_{Sp}$-$LUMO_P$**	-0.2010	-0.2216	-0.2044	-0.2011	-0.1995	-0.2028	-0.1980	-0.2056	-0.2197	-0.2028	-0.2228	-0.2058	-0.2211	-0.2065

$HOMO_P$: Highest Occupied Molecular Orbital of protein; $LUMO_P$: Lowest Unoccupied Molecular Orbital of protein; $LUMO_{Sp}$: Lowest Unoccupied Molecular Orbital of spirostan; $HOMO_{Sp}$: Highest Occupied Molecular Orbital of spirostan.

Table S5. List of designed spirostans.

Sp	C-2	C-3	C4	C-5	C-6	C-11	C-12	C-17	C-25
**1**	H	β OH	H	Δ	Δ	H	H	H	*R*
**2**	H	α OH	H	Δ	Δ	H	H	H	*R*
**3**	H	β OH	H	Δ	Δ	H	H	H	*S*
**4**	H	α OH	H	Δ	Δ	H	H	H	*S*
**5**	H	α OH	H	α H	H	H	H	H	*R*
**6**	H	β OH	H	α H	H	H	H	H	*R*
**7**	H	α OH	H	α H	H	H	H	H	*S*
**8**	H	β OH	H	α H	H	H	H	H	*S*
**9**	H	α OH	H	β H	H	H	H	H	*R*
**10**	H	β OH	H	β H	H	H	H	H	*R*
**11**	H	α OH	H	β H	H	H	H	H	*S*
**12**	H	β OH	H	β H	H	H	H	H	*S*
**13**	H	α OH	H	Δ	Δ	H	O	H	*R*
**14**	H	α OH	H	Δ	Δ	α OH	H	H	*R*
**15**	H	α OH	H	Δ	Δ	β OH	H	H	*R*
**16**	H	α OH	H	Δ	Δ	O	H	H	*R*
**17**	α OH	α OH	H	Δ	Δ	H	H	H	*R*
**18**	β OH	α OH	H	Δ	Δ	H	H	H	*R*
**19**	H	α OH	H	Δ	Δ	H	H	OH	*R*
**20**	H	β OH	H	Δ	Δ	H	O	H	*R*
**21**	H	β OH	H	Δ	Δ	α OH	H	H	*R*
**22**	H	β OH	H	Δ	Δ	β OH	H	H	*R*
**23**	H	β OH	H	Δ	Δ	O	H	H	*R*
**24**	α OH	β OH	H	Δ	Δ	H	H	H	*R*

**25**	*β* OH	*β* OH	H	Δ	Δ	H	H	H	*R*
**26**	H	*β* OH	H	Δ	Δ	H	H	OH	*R*
**27**	H	*α* OH	H	Δ	Δ	H	O	H	*S*
**28**	H	*α* OH	H	Δ	Δ	*α* OH	H	H	*S*
**29**	H	*α* OH	H	Δ	Δ	*β* OH	H	H	*S*
**30**	H	*α* OH	H	Δ	Δ	O	H	H	*S*
**31**	*α* OH	*α* OH	H	Δ	Δ	H	H	H	*S*
**32**	*β* OH	*α* OH	H	Δ	Δ	H	H	H	*S*
**33**	H	*α* OH	H	Δ	Δ	H	H	OH	*S*
**34**	H	*β* OH	H	Δ	Δ	H	O	H	*S*
**35**	H	*β* OH	H	Δ	Δ	*α* OH	H	H	*S*
**36**	H	*β* OH	H	Δ	Δ	*β* OH	H	H	*S*
**37**	H	*β* OH	H	Δ	Δ	O	H	H	*S*
**38**	*α* OH	*β* OH	H	Δ	Δ	H	H	H	*S*
**39**	*β* OH	*β* OH	H	Δ	Δ	H	H	H	*S*
**40**	H	*β* OH	H	Δ	Δ	H	H	OH	*S*
**41**	H	*α* OH	H	*α* H	H	H	O	H	*R*
**42**	H	*α* OH	H	*α* H	H	*α* OH	H	H	*R*
**43**	H	*α* OH	H	*α* H	H	*β* OH	H	H	*R*
**44**	H	*α* OH	H	*α* H	H	O	H	H	*R*
**45**	*α* OH	*α* OH	H	*α* H	H	H	H	H	*R*
**46**	*β* OH	*α* OH	H	*α* H	H	H	H	H	*R*
**47**	H	*α* OH	H	*α* H	H	H	H	OH	*R*
**48**	H	*β* OH	H	*α* H	H	H	O	H	*R*
**49**	H	*β* OH	H	*α* H	H	*α* OH	H	H	*R*
**50**	H	*β* OH	H	*α* H	H	*β* OH	H	H	*R*

**51**	H	*β* OH	H	*α* H	H	O	H	H	*R*
**52**	*α* OH	*β* OH	H	*α* H	H	H	H	H	*R*
**53**	*β* OH	*β* OH	H	*α* H	H	H	H	H	*R*
**54**	H	*β* OH	H	*α* H	H	H	H	OH	*R*
**55**	H	*α* OH	H	*α* H	H	H	O	H	*S*
**56**	H	*α* OH	H	*α* H	H	*α* OH	H	H	*S*
**57**	H	*α* OH	H	*α* H	H	*β* OH	H	H	*S*
**58**	H	*α* OH	H	*α* H	H	O	H	H	*S*
**59**	*α* OH	*α* OH	H	*α* H	H	H	H	H	*S*
**60**	*β* OH	*α* OH	H	*α* H	H	H	H	H	*S*
**61**	H	*α* OH	H	*α* H	H	H	H	OH	*S*
**62**	H	*β* OH	H	*α* H	H	H	O	H	*S*
**63**	H	*β* OH	H	*α* H	H	*α* OH	H	H	*S*
**64**	H	*β* OH	H	*α* H	H	*β* OH	H	H	*S*
**65**	H	*β* OH	H	*α* H	H	O	H	H	*S*
**66**	*α* OH	*β* OH	H	*α* H	H	H	H	H	*S*
**67**	*β* OH	*β* OH	H	*α* H	H	H	H	H	*S*
**68**	H	*β* OH	H	*α* H	H	H	H	OH	*S*
**69**	H	*α* OH	H	*β* H	H	H	O	H	*R*
**70**	H	*α* OH	H	*β* H	H	*α* OH	H	H	*R*
**71**	H	*α* OH	H	*β* H	H	*β* OH	H	H	*R*
**72**	H	*α* OH	H	*β* H	H	O	H	H	*R*
**73**	*α* OH	*α* OH	H	*β* H	H	H	H	H	*R*
**74**	*β* OH	*α* OH	H	*β* H	H	H	H	H	*R*
**75**	H	*α* OH	H	*β* H	H	H	H	OH	*R*
**76**	H	*β* OH	H	*β* H	H	H	O	H	*R*

**77**	H	*β* OH	H	*β* H	H	*α* OH	H	H	*R*
**78**	H	*β* OH	H	*β* H	H	*β* OH	H	H	*R*
**79**	H	*β* OH	H	*β* H	H	O	H	H	*R*
**80**	*α* OH	*β* OH	H	*β* H	H	H	H	H	*R*
**81**	*β* OH	*β* OH	H	*β* H	H	H	H	H	*R*
**82**	H	*β* OH	H	*β* H	H	H	H	OH	*R*
**83**	H	*α* OH	H	*β* H	H	H	O	H	*S*
**84**	H	*α* OH	H	*β* H	H	*α* OH	H	H	*S*
**85**	H	*α* OH	H	*β* H	H	*β* OH	H	H	*S*
**86**	H	*α* OH	H	*β* H	H	O	H	H	*S*
**87**	*α* OH	*α* OH	H	*β* H	H	H	H	H	*S*
**88**	*β* OH	*α* OH	H	*β* H	H	H	H	H	*S*
**89**	H	*α* OH	H	*β* H	H	H	H	OH	*S*
**90**	H	*β* OH	H	*β* H	H	H	O	H	*S*
**91**	H	*β* OH	H	*β* H	H	*α* OH	H	H	*S*
**92**	H	*β* OH	H	*β* H	H	*β* OH	H	H	*S*
**93**	H	*β* OH	H	*β* H	H	O	H	H	*S*
**94**	*α* OH	*β* OH	H	*β* H	H	H	H	H	*S*
**95**	*β* OH	*β* OH	H	*β* H	H	H	H	H	*S*
**96**	H	*β* OH	H	*β* H	H	H	H	OH	*S*
**97**	*α* OH	*β* OH	H	Δ	Δ	H	O	H	*R*
**98**	H	*β* OH	H	*α* H	O	H	H	H	*R*
**99**	H	*α* OH	H	*α* H	O	H	H	H	*R*
**100**	H	*α* OH	H	*α* H	O	H	H	H	*S*
**101**	H	*β* OH	H	*α* H	O	H	H	H	*S*
**102**	H	*β* OH	H	*β* H	O	H	H	H	*R*

**103**	H	α OH	H	β H	O	H	H	H	*R*
**104**	H	α OH	H	β H	O	H	H	H	*S*
**105**	H	β OH	H	β H	O	H	H	H	*S*
**106**	H	β OH	H	α H	α OH	H	H	H	*R*
**107**	H	α OH	H	α H	α OH	H	H	H	*R*
**108**	H	α OH	H	α H	α OH	H	H	H	*S*
**109**	H	β OH	H	α H	α OH	H	H	H	*S*
**110**	H	β OH	H	α H	β OH	H	H	H	*R*
**111**	H	α OH	H	α H	β OH	H	H	H	*R*
**112**	H	α OH	H	α H	β OH	H	H	H	*S*
**113**	H	β OH	H	α H	β OH	H	H	H	*S*
**114**	H	β OH	H	β H	α OH	H	H	H	*R*
**115**	H	α OH	H	β H	α OH	H	H	H	*R*
**116**	H	α OH	H	β H	α OH	H	H	H	*S*
**117**	H	β OH	H	β H	α OH	H	H	H	*S*
**118**	H	β OH	H	β H	β OH	H	H	H	*R*
**119**	H	α OH	H	β H	β OH	H	H	H	*R*
**120**	H	α OH	H	β H	β OH	H	H	H	*S*
**121**	H	β OH	H	β H	β OH	H	H	H	*S*
**122**	H	β OH	H	α OH	O	H	H	H	*R*
**123**	H	α OH	H	α OH	O	H	H	H	*R*
**124**	H	α OH	H	α OH	O	H	H	H	*S*
**125**	H	β OH	H	α OH	O	H	H	H	*S*
**126**	H	β OH	H	α OH	α OH	H	H	H	*R*
**127**	H	α OH	H	α OH	α OH	H	H	H	*R*
**128**	H	α OH	H	α OH	α OH	H	H	H	*S*

**129**	H	β OH	H	α OH	α OH	H	H	H	*S*
**130**	H	β OH	H	α OH	β OH	H	H	H	*R*
**131**	H	α OH	H	α OH	β OH	H	H	H	*R*
**132**	H	α OH	H	α OH	β OH	H	H	H	*S*
**133**	H	β OH	H	α OH	β OH	H	H	H	*S*
**134**	H	O	Δ	NP	H	H	H	H	*R*
**135**	H	O	Δ	NP	H	H	H	H	*S*
**136**	H	O	Δ	NP	α OH	H	H	H	*R*
**137**	H	O	Δ	NP	α OH	H	H	H	*S*
**138**	H	O	Δ	NP	β OH	H	H	H	*R*
**139**	H	O	Δ	NP	β OH	H	H	H	*S*
**140**	H	O	Δ	NP	O	H	H	H	*R*
**141**	H	O	Δ	NP	O	H	H	H	*S*
**142**	α OH	O	Δ	NP	H	H	H	H	*R*
**143**	α OH	O	Δ	NP	H	H	H	H	*S*
**144**	β OH	O	Δ	NP	H	H	H	H	*R*
**145**	β OH	O	Δ	NP	H	H	H	H	*S*
**146**	H	O	Δ	NP	H	α OH	H	H	*R*
**147**	H	O	Δ	NP	H	α OH	H	H	*S*
**148**	H	O	Δ	NP	H	β OH	H	H	*R*
**149**	H	O	Δ	NP	H	β OH	H	H	*S*
**150**	H	O	Δ	NP	H	O	H	H	*R*
**151**	H	O	Δ	NP	H	O	H	H	*S*
**152**	H	O	Δ	NP	H	H	O	H	*R*
**153**	H	O	Δ	NP	H	H	O	H	*S*
**154**	H	O	Δ	NP	H	H	H	OH	*R*

**155**	H	O	Δ	NP	H	H	H	OH	*S*

a)

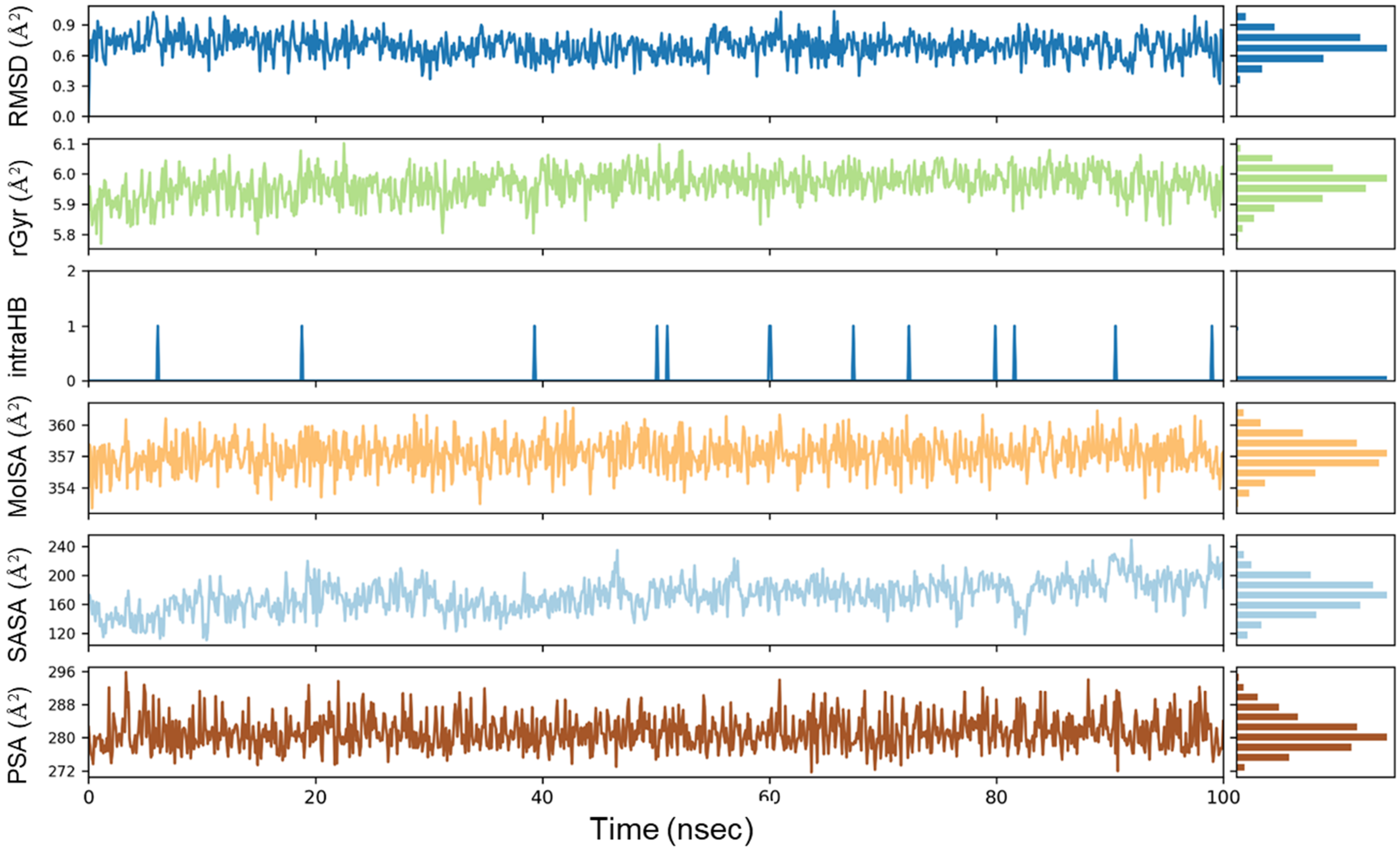
RMSD (Å²)
0.0
0.3
0.6
0.9
rGyr (Å²)
5.8
5.9
6.0
6.1
intraHB
0
1
2
MolSA (Å²)
354
357
360
SASA (Å²)
120
160
200
240
PSA (Å²)
272
280
288
296
0
20
40
60
80
100
Time (nsec)

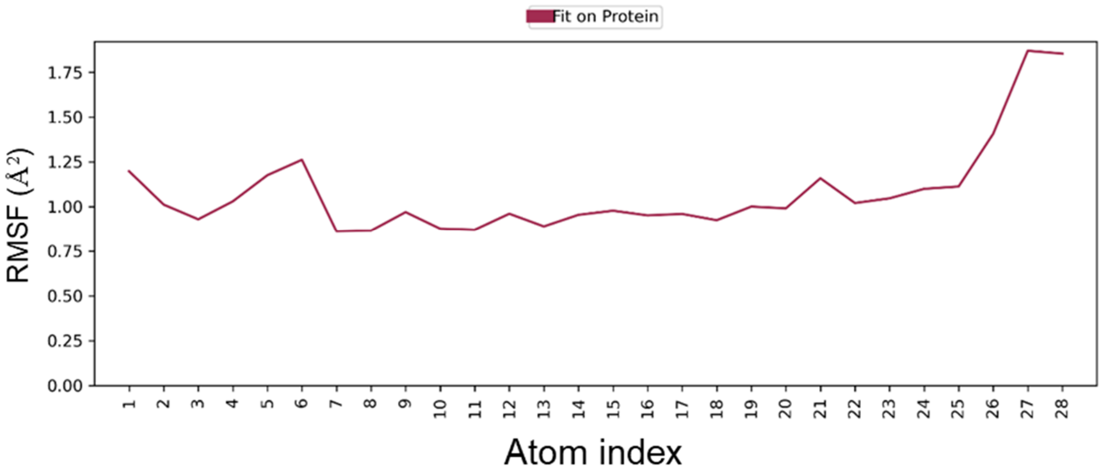
Fit on Protein
1.75
1.50
1.25
1.00
0.75
0.50
0.25
0.00
RMSF (Å²)
1
2
3
4
5
6
7
8
9
10
11
12
13
14
15
16
17
18
19
20
21
22
23
24
25
26
27
28
Atom index

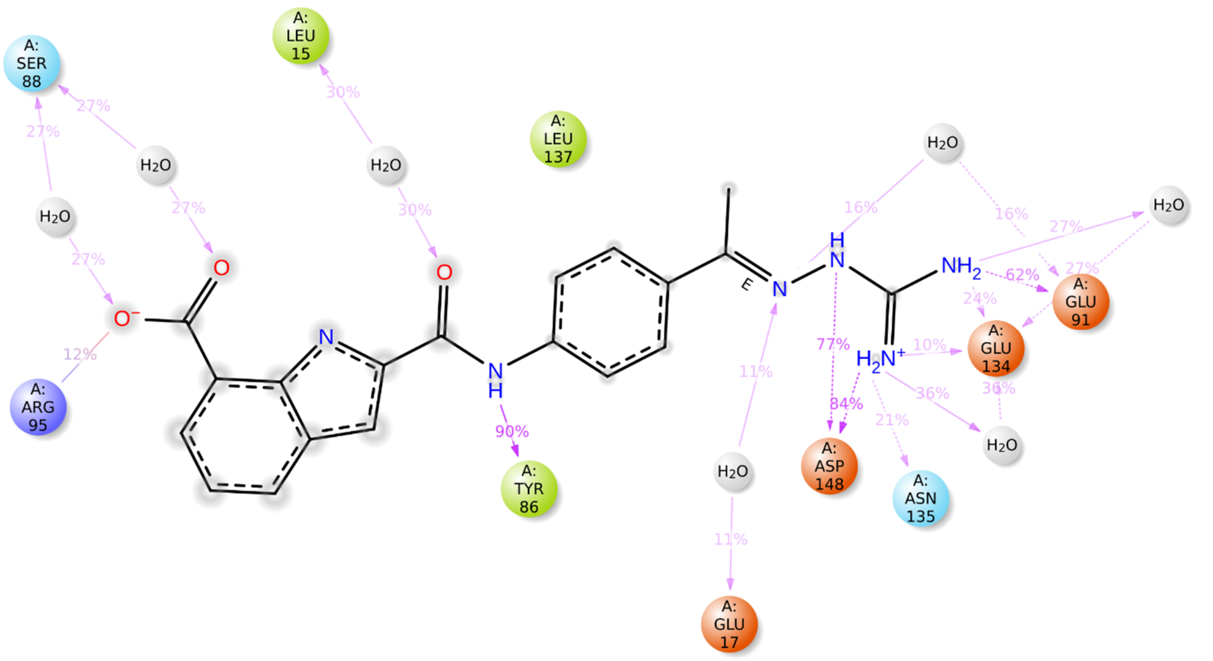

A: SER 88
A: LEU 15
A: LEU 137
A: ARG 95
A: TYR 86
A: GLU 17
A: ASP 148
A: ASN 135
A: GLU 134
A: GLU 91
H_2O
27%
30%
12%
90%
11%
16%
77%
84%
21%
36%
10%
24%
62%

b)

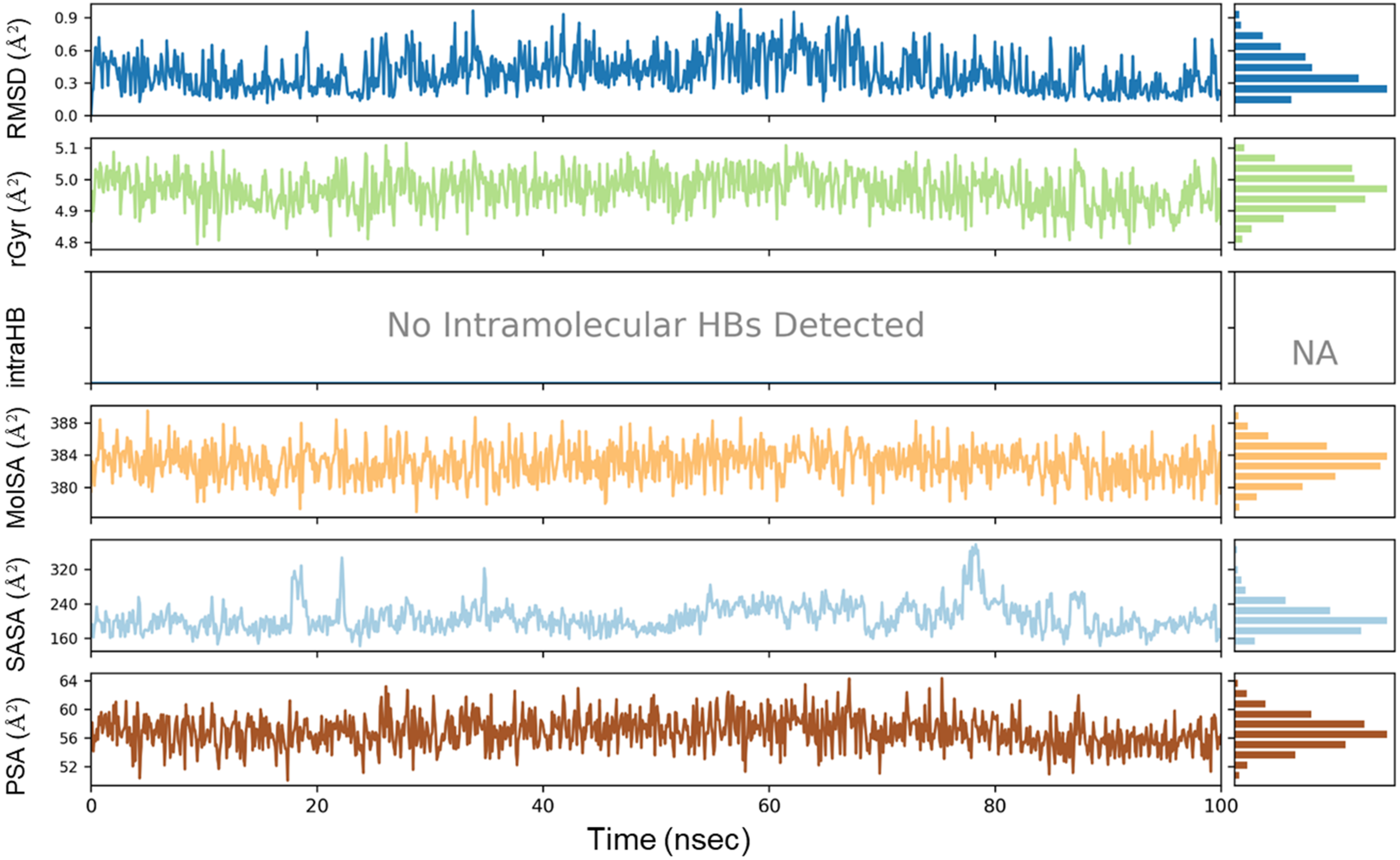

RMSD (Å^2)
0.9
0.6
0.3
0.0
rGyr (Å^2)
5.1
5.0
4.9
4.8
intraHB
No Intramolecular HBs Detected
NA
MolSA (Å^2)
388
384
380
SASA (Å^2)
320
240
160
PSA (Å^2)
64
60
56
52
0
20
40
60
80
100
Time (nsec)

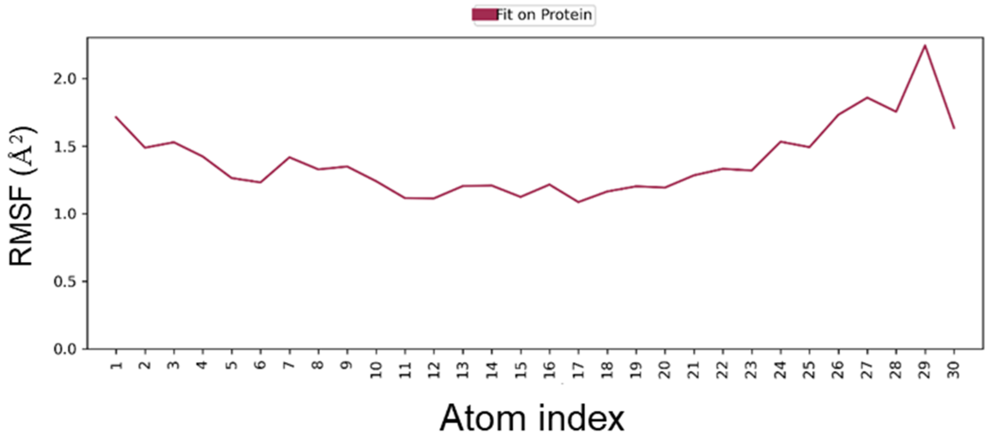
Fit on Protein
RMSF (Å²)
2.0
1.5
1.0
0.5
0.0
1 2 3 4 5 6 7 8 9 10 11 12 13 14 15 16 17 18 19 20 21 22 23 24 25 26 27 28 29 30
Atom index

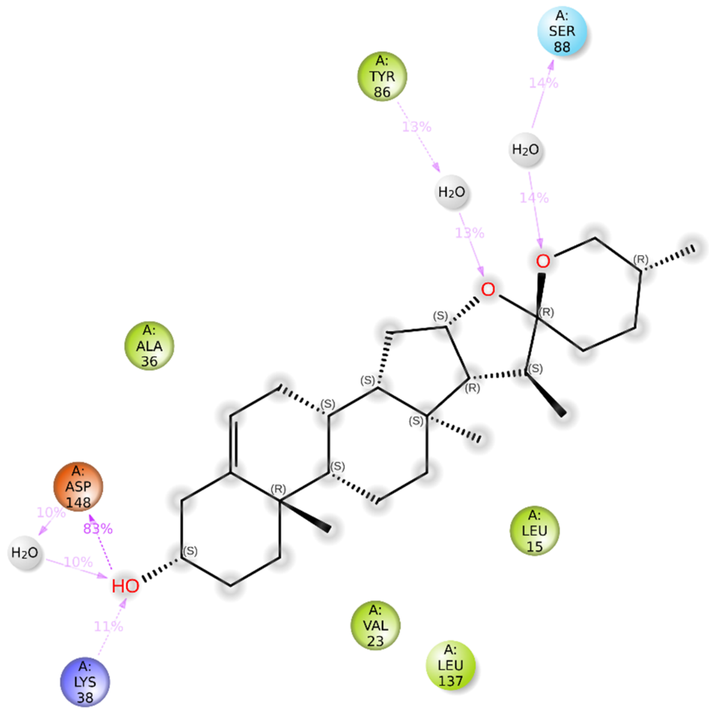
A:
SER
88
A:
TYR
86
14%
13%
H_2O
H_2O
14%
13%
A:
ALA
36
A:
ASP
148
10%
83%
H_2O
10%
HO
11%
A:
LYS
38
A:
LEU
15
A:
VAL
23
A:
LEU
137

c)

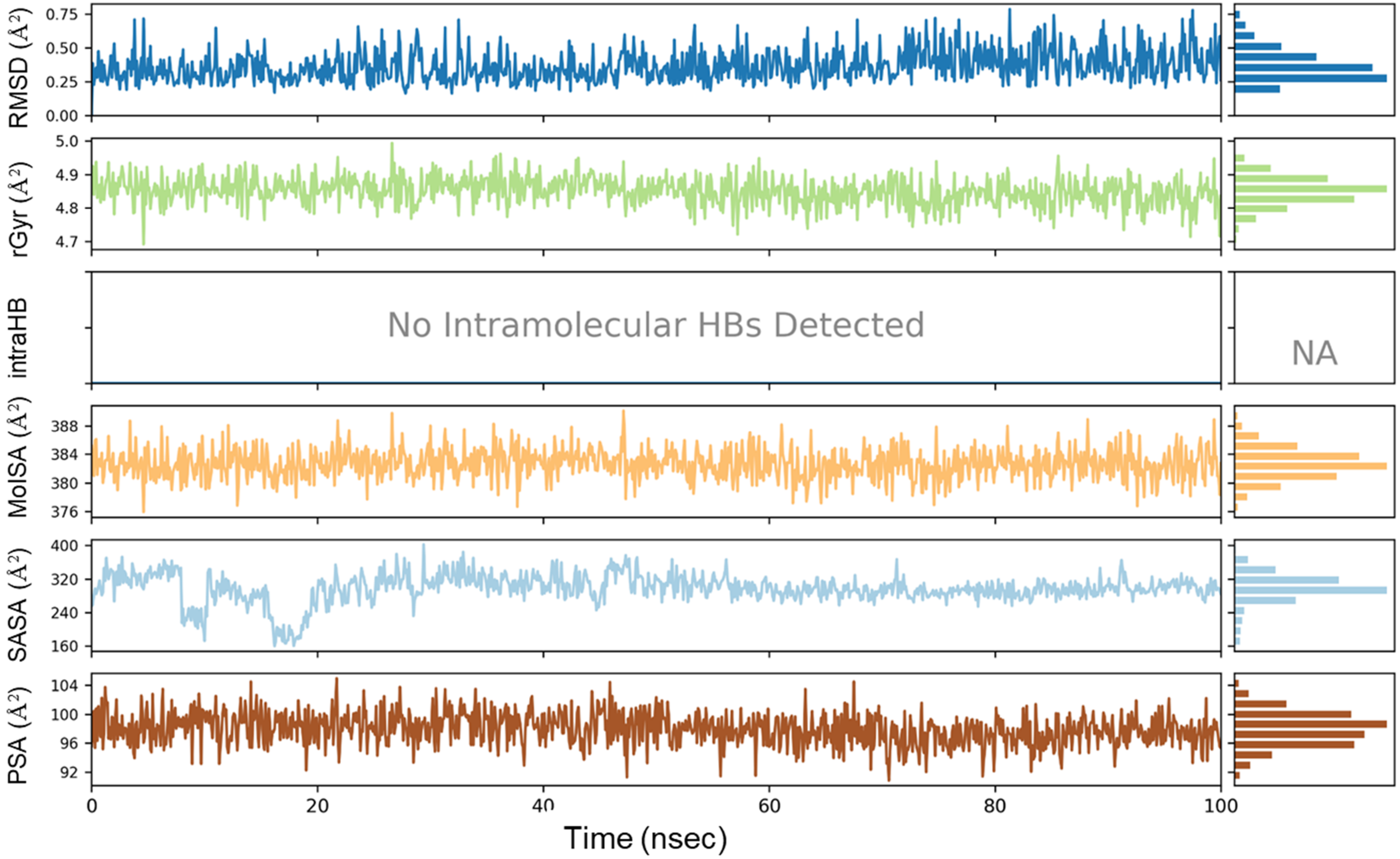

RMSD (Å²)
0.75
0.50
0.25
0.00
rGyr (Å²)
5.0
4.9
4.8
4.7
intraHB
No Intramolecular HBs Detected
NA
MolSA (Å²)
388
384
380
376
SASA (Å²)
400
320
240
160
PSA (Å²)
104
100
96
92
0
20
40
60
80
100
Time (nsec)

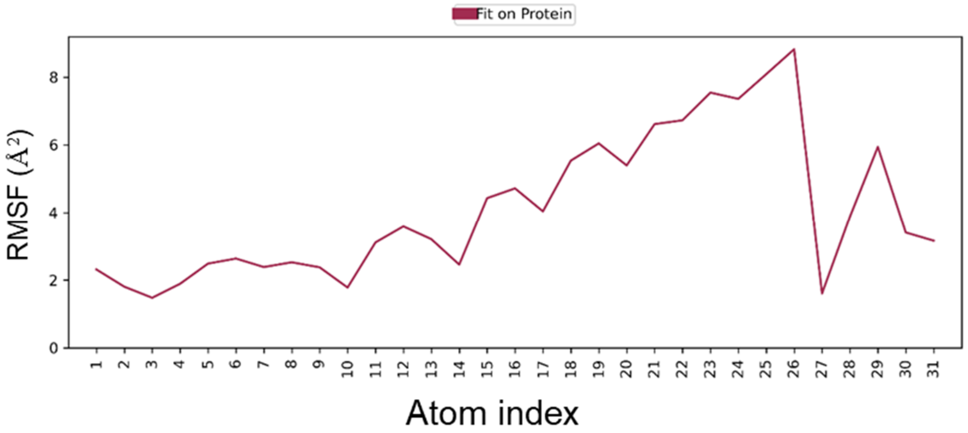

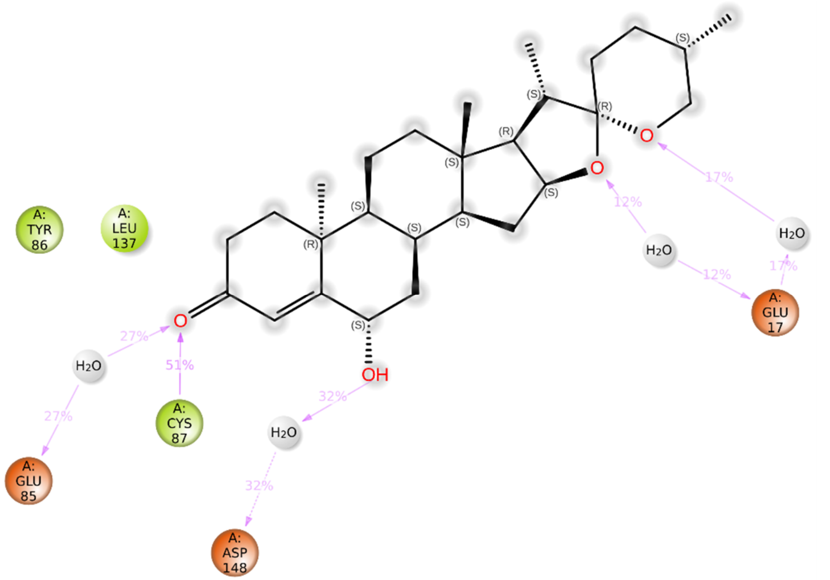

Figure S1: Ligand-protein contacts, stability, RMSF per ligand, SASA, PSA, torsions and contacts as a function of time with CHK1 for a) CCT241533; b) Sp1; c) Sp137.

a)

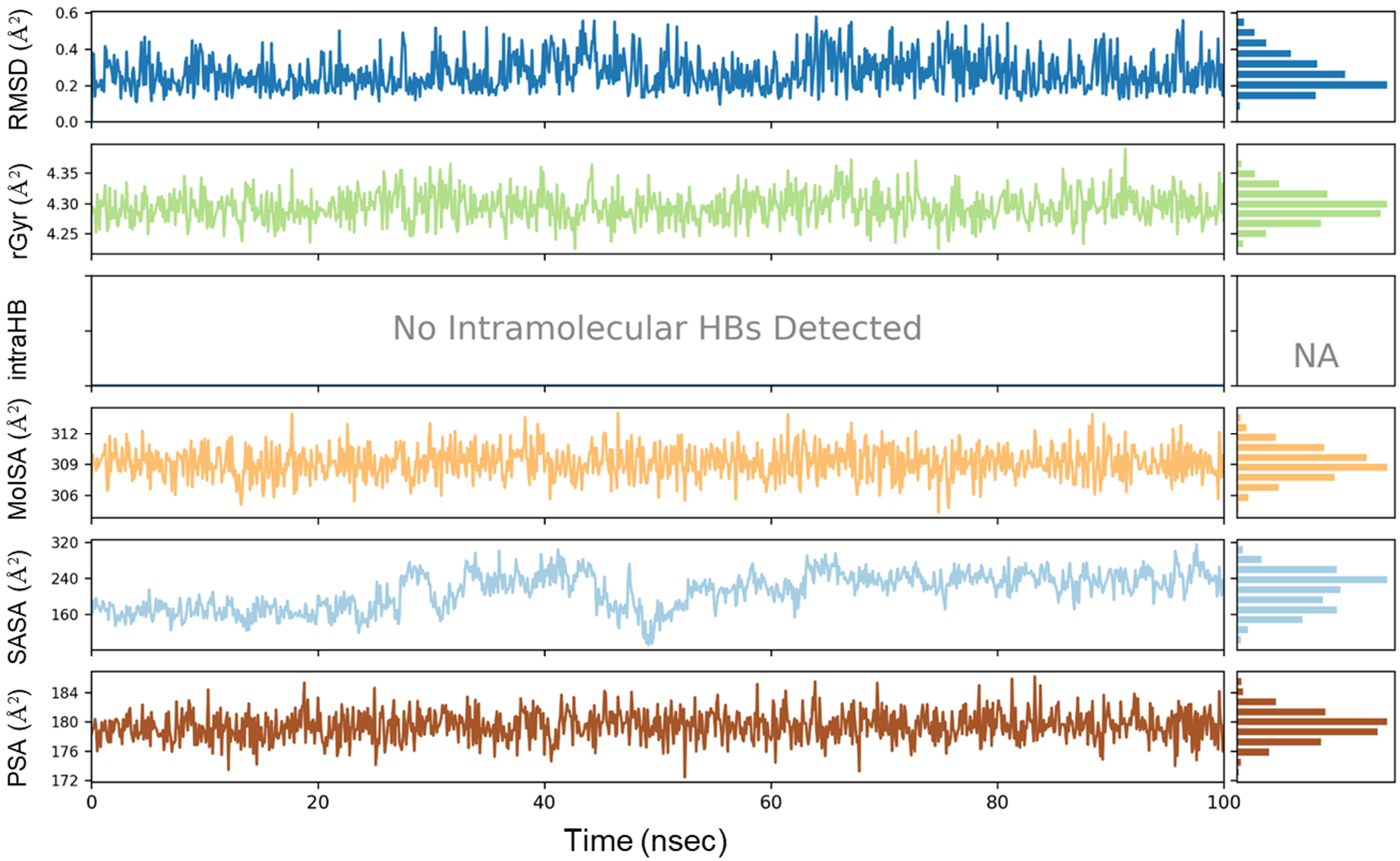
RMSD (Å²)
0.6
0.4
0.2
0.0
rGyr (Å²)
4.35
4.30
4.25
intraHB
No Intramolecular HBs Detected
NA
MolSA (Å²)
312
309
306
SASA (Å²)
320
240
160
PSA (Å²)
184
180
176
172
0
20
40
60
80
100
Time (nsec)

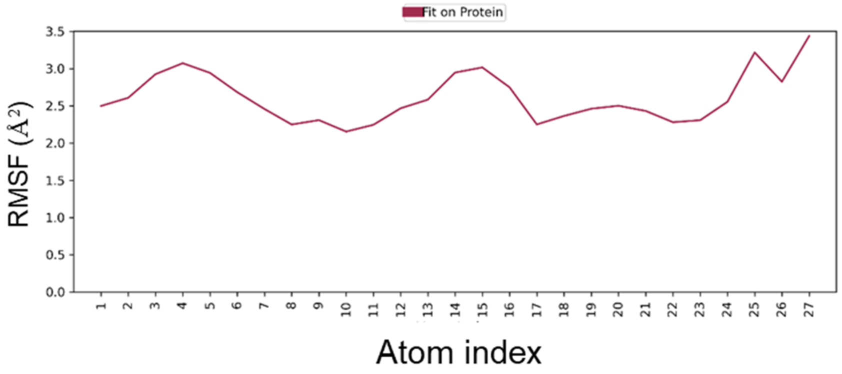
Fit on Protein
RMSF (Å²)
Atom index

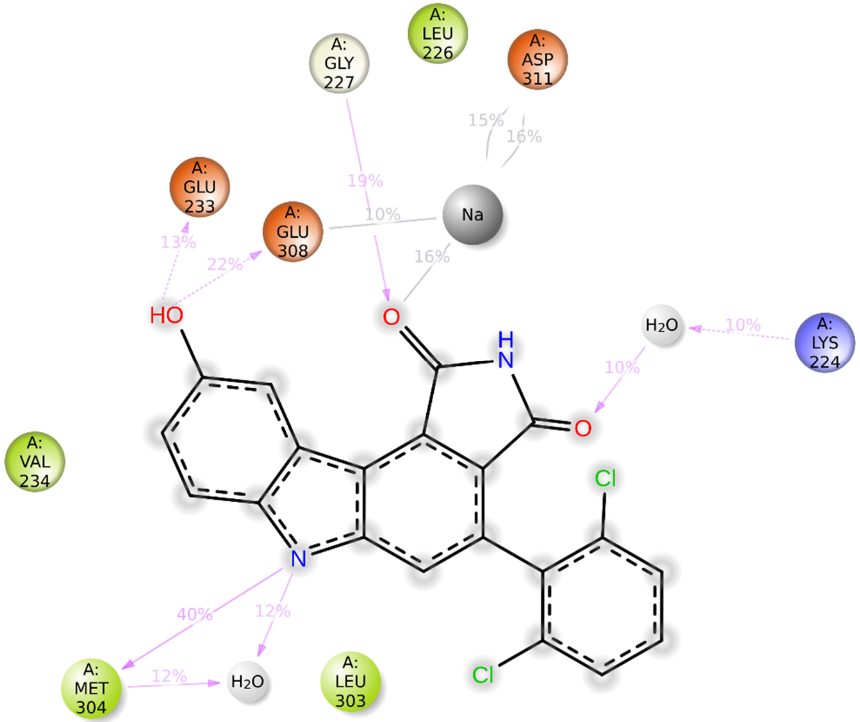
A: GLY 227
A: LEU 226
A: ASP 311
A: GLU 233
A: GLU 308
Na
A: LYS 224
A: VAL 234
A: MET 304
A: LEU 303
HO
H2O
Cl
19%
10%
15%
16%
13%
22%
40%
12%

b)

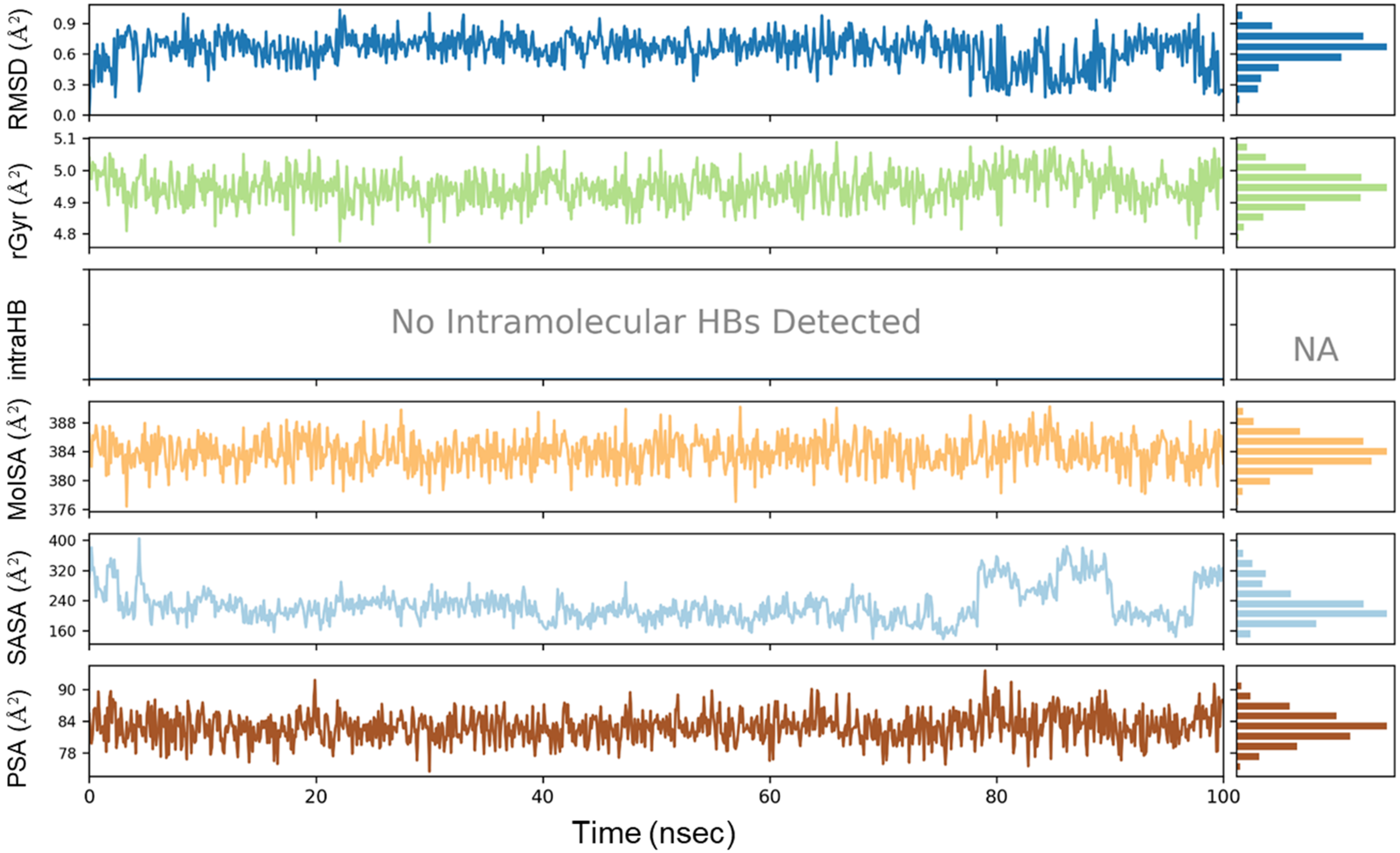
RMSD (Å²)
0.0
0.3
0.6
0.9
rGyr (Å²)
4.8
4.9
5.0
5.1
intraHB
No Intramolecular HBs Detected
NA
MolSA (Å²)
376
380
384
388
SASA (Å²)
160
240
320
400
PSA (Å²)
78
84
90
0
20
40
60
80
100
Time (nsec)

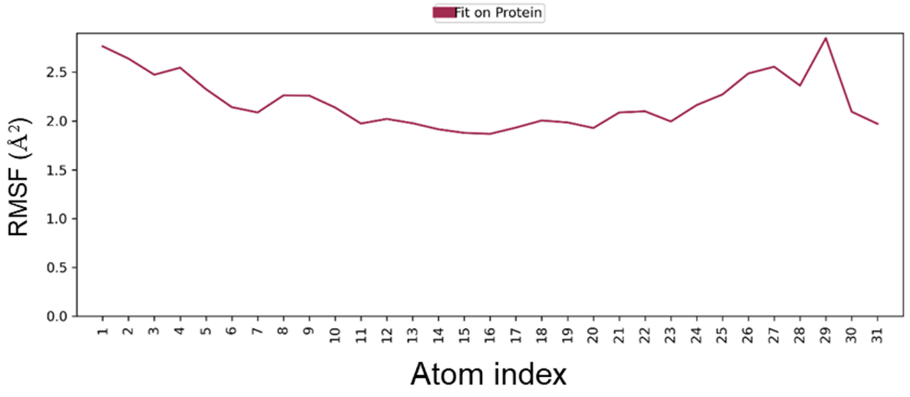
Fit on Protein
RMSF (Å²)
Atom index

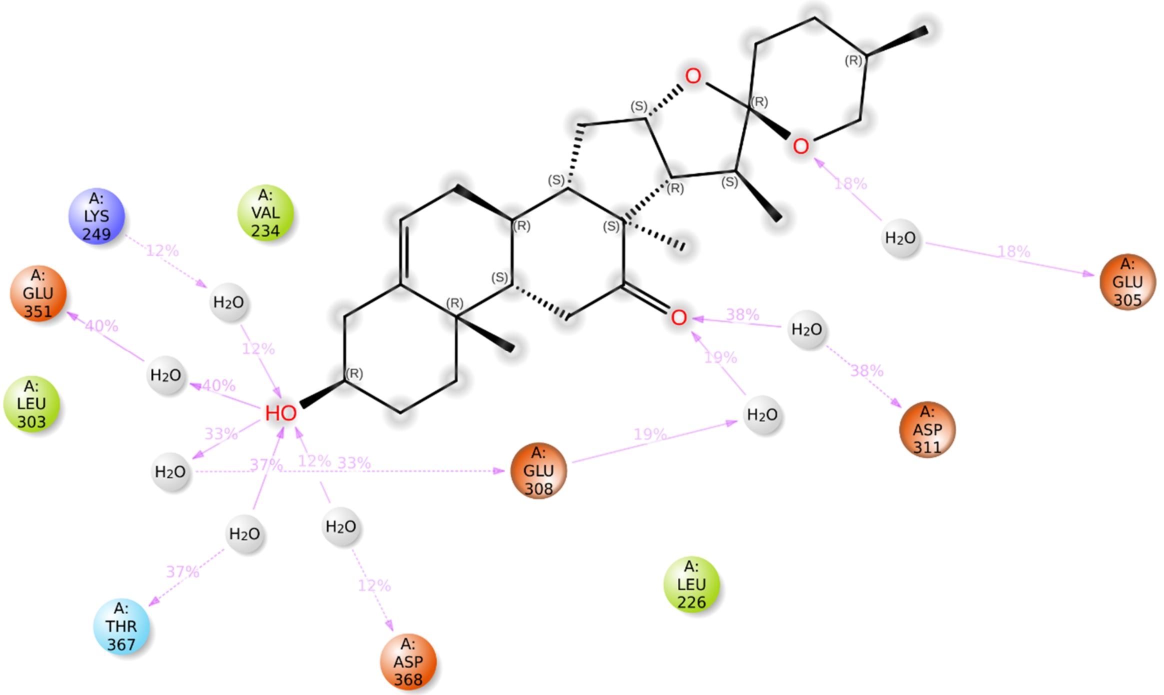
A: LYS 249
A: VAL 234
A: GLU 351
A: LEU 303
A: THR 367
A: ASP 368
A: GLU 308
A: LEU 226
A: ASP 311
A: GLU 305
HO
O
H2O
12%
40%
33%
37%
19%
38%
18%

c)

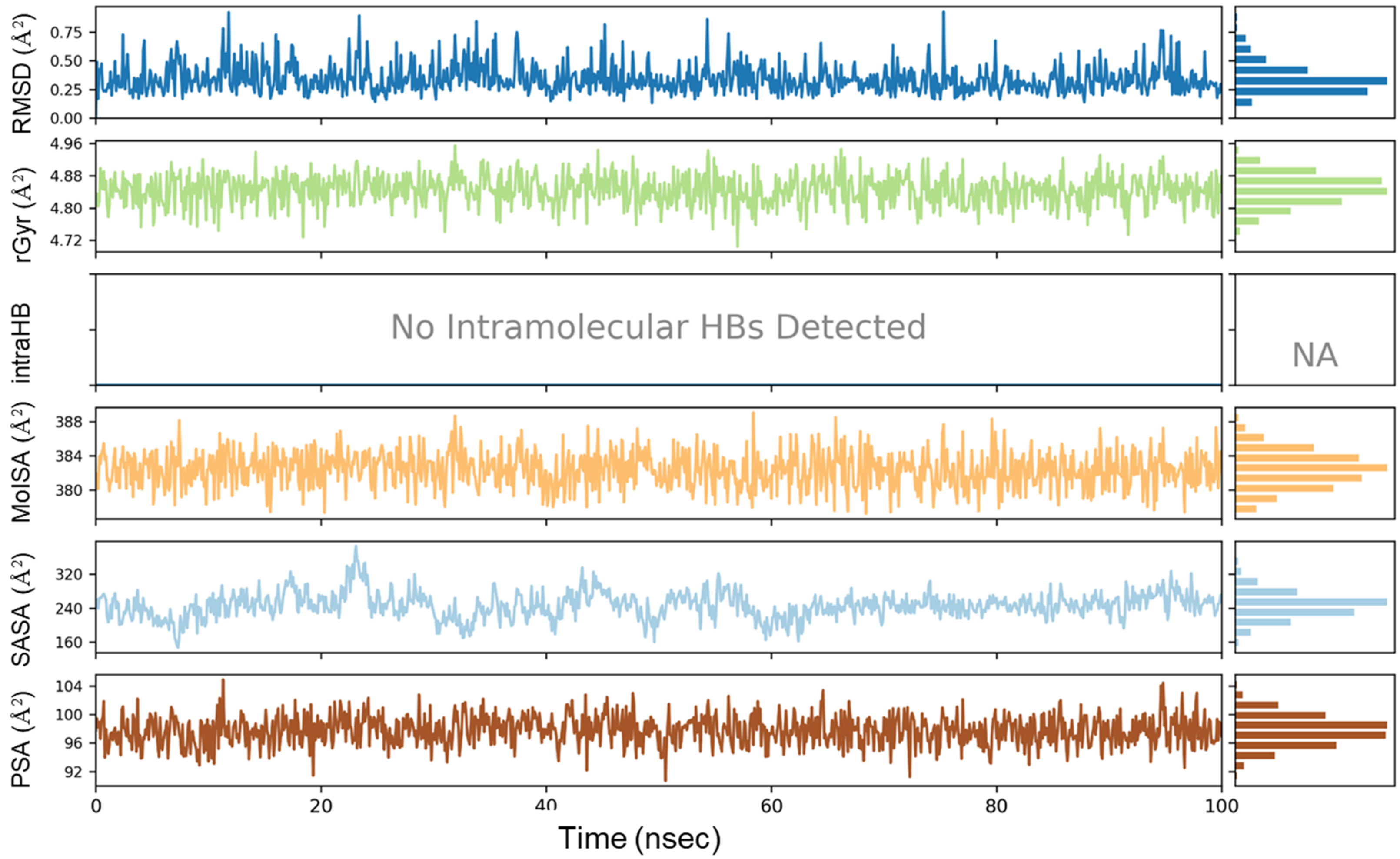
RMSD (Å²)
0.75
0.50
0.25
0.00
rGyr (Å²)
4.96
4.88
4.80
4.72
intraHB
No Intramolecular HBs Detected
NA
MolSA (Å²)
388
384
380
SASA (Å²)
320
240
160
PSA (Å²)
104
100
96
92
0
20
40
60
80
100
Time (nsec)

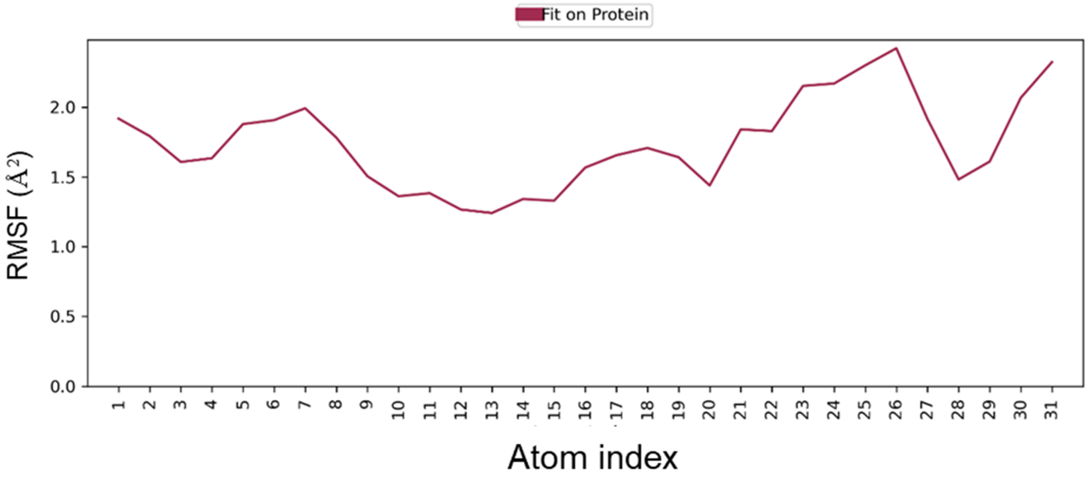

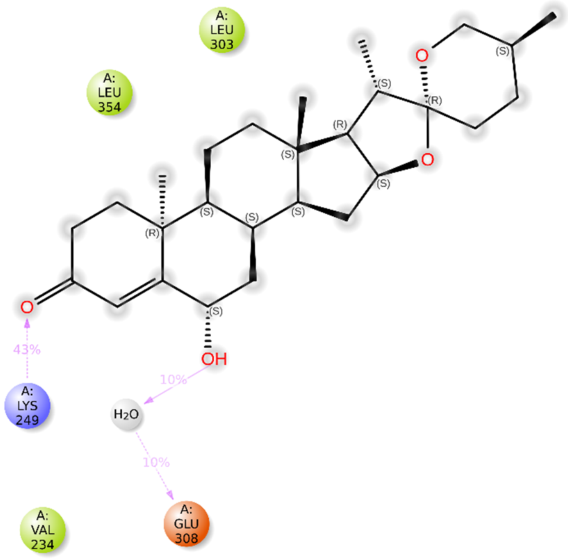

Figure S2: Ligand-protein contacts, stability, RMSF per ligand, SASA, PSA, torsions and contacts as a function of time with CHK2 for a) PD407824; b) Sp13; c) Sp137.

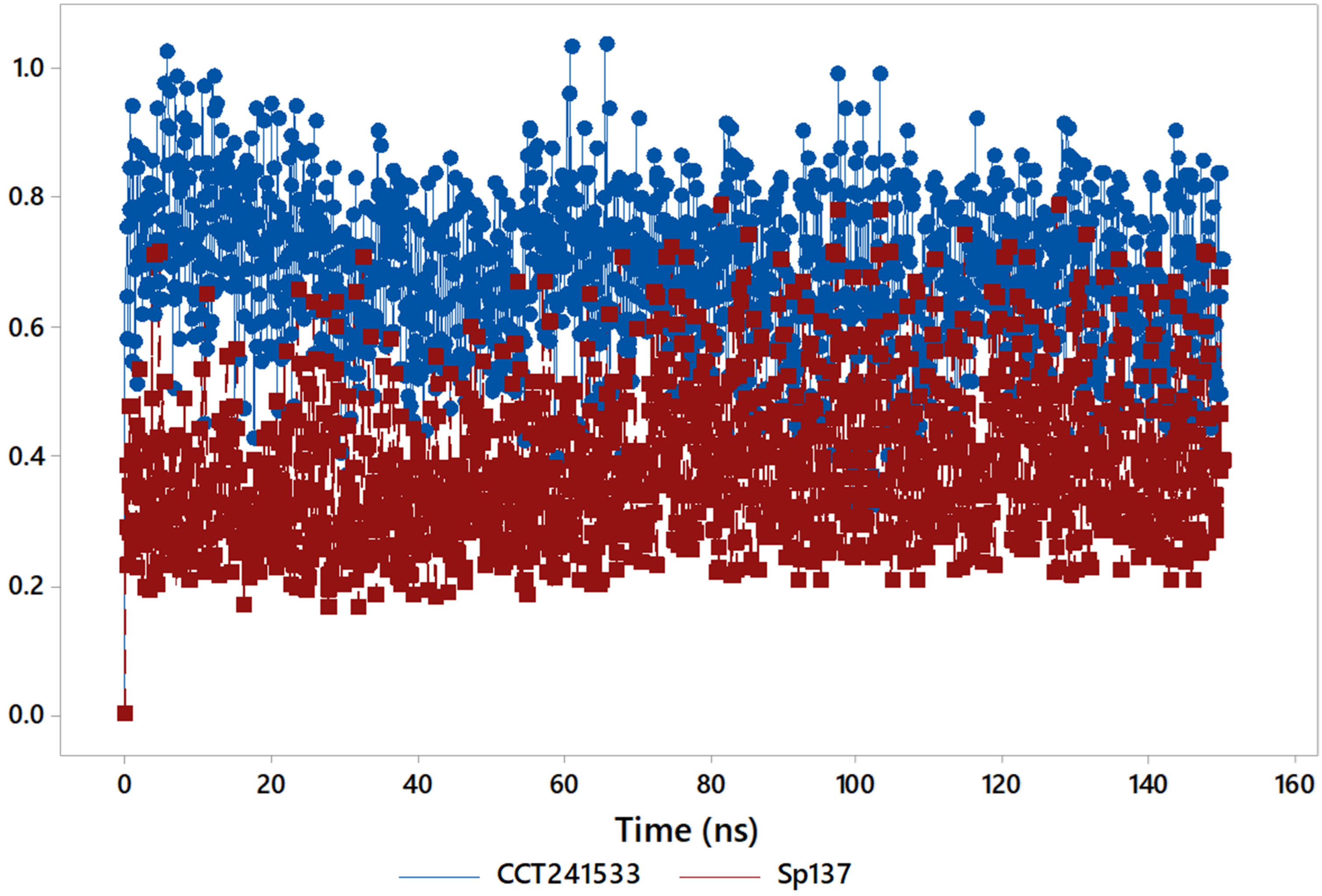

Figure S3. Representative RMSD Ligand-Protein at 150 ns in CHK1 to CCT241533 and Sp137.
